# Frataxin deficiency increases cyclooxygenase 2 and prostaglandins in cell and animal models of Friedreich's ataxia

**DOI:** 10.1093/hmg/ddu407

**Published:** 2014-08-07

**Authors:** Genki Hayashi, Yan Shen, Theresa L. Pedersen, John W. Newman, Mark Pook, Gino Cortopassi

**Affiliations:** 1 Department ofMolecular Biosciences and; 2 Department of Nutrition, University of California, Davis, CA 95616, USA; 3 USDA, ARS, Western Human Nutrition Research Center, 430 West Health Sciences Dr, Davis, CA 95616, USA; 4 West Coast Metabolomics Center, University of California Davis Genome Center, Davis, CA 95616, USA and; 5 Department of Biosciences, Brunel University, Uxbridge, Middlesex, UK

## Abstract

An inherited deficiency of the mitochondrial protein frataxin causes Friedreich's ataxia (FRDA); the mechanism by which this deficiency triggers neuro- and cardio-degeneration is unclear. Microarrays of neural tissue of animal models of the disease showed decreases in antioxidant genes, and increases in inflammatory genes. Cyclooxygenase (COX)-derived oxylipins are important mediators of inflammation. We measured oxylipin levels using tandem mass spectrometry and ELISAs in multiple cell and animal models of FRDA. Mass spectrometry revealed increases in concentrations of prostaglandins, thromboxane B2, 15-HETE and 11-HETE in cerebellar samples of knockin knockout mice. One possible explanation for the elevated oxylipins is that frataxin deficiency results in increased COX activity. While constitutive COX1 was unchanged, inducible COX2 expression was elevated over 1.35-fold (*P* < 0.05) in two Friedreich's mouse models and Friedreich's lymphocytes. Consistent with higher COX2 expression, its activity was also increased by 58% over controls. COX2 expression is driven by multiple transcription factors, including activator protein 1 and cAMP response element-binding protein, both of which were elevated over 1.52-fold in cerebella. Taken together, the results support the hypothesis that reduced expression of frataxin leads to elevation of COX2-mediated oxylipin synthesis stimulated by increases in transcription factors that respond to increased reactive oxygen species. These findings support a neuroinflammatory mechanism in FRDA, which has both pathomechanistic and therapeutic implications.

## INTRODUCTION

Friedreich's ataxia (FRDA) is the most common autosomal recessive ataxia affecting ∼1:40 000 individuals (1). The disease is characterized by the neurodegeneration of dentate nucleus of the cerebellum and demyelination in spinocerebellar dorsal root ganglion neurons as well as hypertrophic cardiomyopathy and diabetes (2–4). Clinical symptoms of FRDA include gait ataxia affecting the motor coordination, weakness and atrophy of the extremities, and loss of lower-limb tendon reflexes. The disease is caused by reduced expression of frataxin gene attributed to the repeat expansion of GAA in the first intron of the gene or point mutation truncating the protein (1,5–7).

Extensive knowledge on the role of oxidative stress in the pathogenesis of FRDA exists including studies involving FRDA patient-derived cells and various mouse models. FRDA patient-derived cells have increased sensitivity to oxidative stress (8,9), and patients were observed to have increased markers of oxidative stress in their blood and urine (10–12). Furthermore, reduced Frataxin appears to reduce the level and or inducibility of Nuclear factor (erythroid-derived 2)-like 2 (NRF2) in cell and animal models of Friedreich's (13,14), which has been demonstrated to have anti-inflammatory effects in other systems patients may impair the induction of early antioxidant defenses resulting in increased cell lethality when under oxidative stress. Consistently, studies involving transgenic mouse models YG8 and YG22, both expressing human frataxin with trinucleotide repeat (GAA)^≤190^ reducing frataxin transcription showed signs of increased oxidative stress in the cerebellum, heart and skeletal muscles (14,15).

The prostaglandin (PG) pathway has been shown to be induced in response to oxidative stress and may be over stimulated by the chronic reactive oxygen species (ROS) elevation in frataxin-deficient models (16). PGs and thromboxanes are a subclass of eicosanoids produced by cyclooxygenase (COX)-mediated conversion of arachidonic acid in response to various stimuli (17–19). Previously we showed that inflammatory eicosanoids including PGs and thromboxane B2 (TXB2) and cytokines rose in frataxin-depleted Schwann cells (20). Consistent with the induction of inflammation in these frataxin-deficient cells, the decreased viability of the frataxin-deficient Schwann cells was rescued by known anti-inflammatory and anti-apoptotic drugs (20). Of the inhibitors, those targeting P38 kinases were most effective rescuers, suggesting a cAMP response element-binding protein (CREB) driven regulation of PG instigating the rescue in cell viability. Along with CREB, transcription factors, nuclear factor kappa-light-chain-enhancer of activated B cells (NFκB) and activator protein 1 (AP1) also regulate the expression of COX2 (21–23).

Inflammatory changes have been noted in the neurodegenerative conditions Alzheimer's disease (24,25), Parkinson's disease (26) and Amyotrophic lateral sclerosis (27,28). Given the previous implications of inflammatory PGs in cell models of FRDA pathogenesis, we investigated COX metabolites in cerebella of FRDA mouse models and patient B-lymphocytes. Significant differences were observed and an underlying mechanism is suggested.

## RESULTS

### Confirmation of frataxin deficiency in mouse and cell models by qRT–PCR

**Figure 1.**
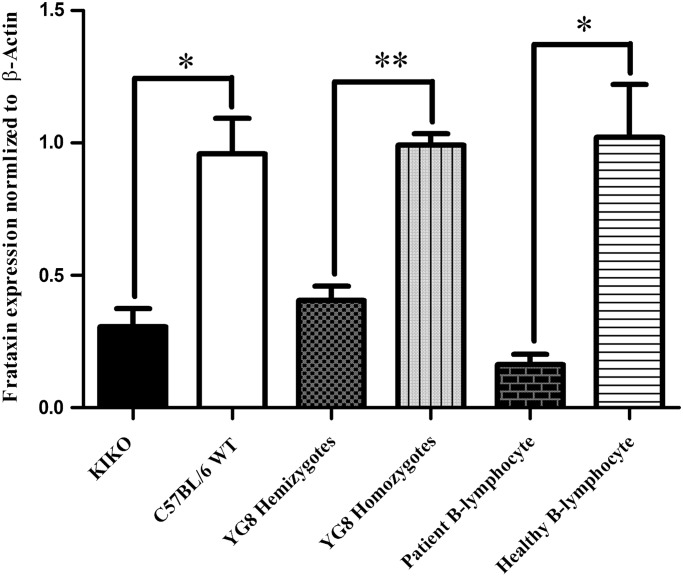
Reduced frataxin expression in frataxin-deficient models analyzed by qRT–PCR. Relative expression was normalized to β-actin and control using ΔΔ^CT^ calculation. Cerebellum of KIKO showed 0.317-fold decreased expression compared with C57Bl/6. Cerebellum of YG8 hemizygotes showed 0.410-fold decreased expression compared with YG8 homozygote. Patient B-lymphocytes showed 0.159-fold decreased expression compared with healthy B-lymphocytes. Bars represent averages ± standard deviations (*n* = 4, *P* < 0.005*, *P* < 0.0005**).

The cerebellar frataxin deficiency of the mouse frataxin knockin-knockout (KIKO) mouse was quantified by qRT–PCR; the animals contained 31% of frataxin compared with wild type (WT) mice as control (*P* > 0.0032, *n* = 4). Similarly, frataxin levels were quantified in the YG8 hemi and homozygote (15), and in cellular models of FRDA; all mutants were significantly deficient in frataxin. The hemizygote mice expressed 41% of frataxin as homozygote (*P* > 0.00013, *n* = 4) and patient-derived B-lymphocytes expressed 15.9% of frataxin compared with healthy B-lymphocytes (*P* > 0.0021, *n* = 4) (Fig 1).

### Elevation of COX-derived oxylipins in frataxin-deficient mice

**Figure 2.**
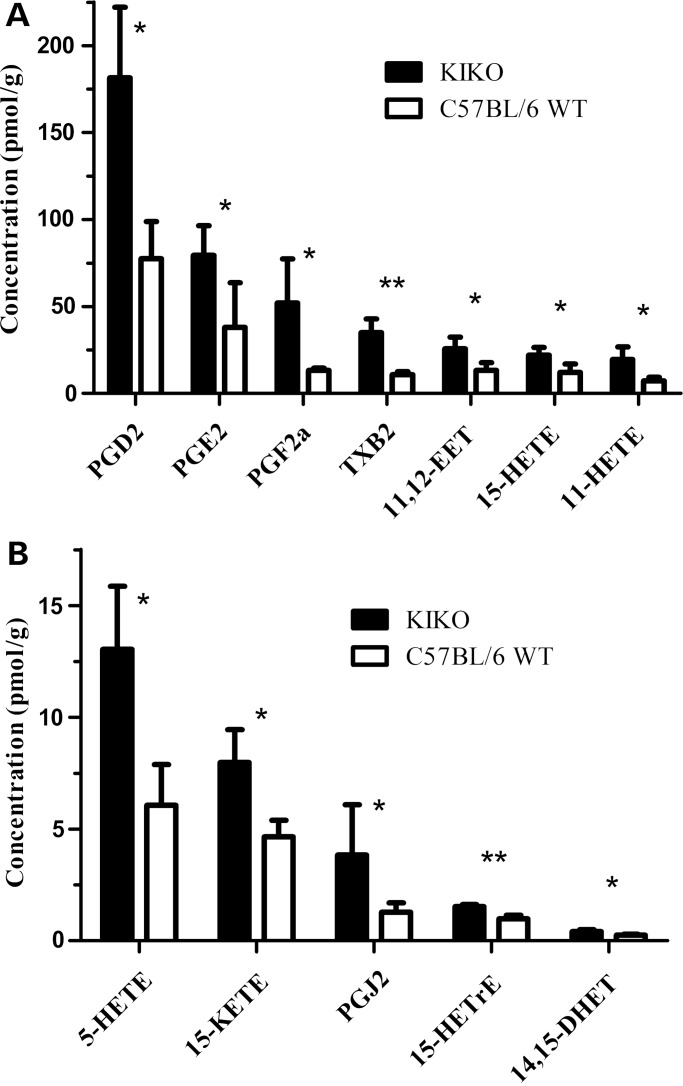
Inflammatory arachidonic metabolites are elevated in cerebellum of frataxin-deficient KIKO mouse model. (**A** and **B**) 12 arachidonic metabolites converted by COX, lipoxygenase and cytochrome P450 were identified to be significantly increased. Grubbs test was used to exclude outliers assuming normal distribution. Bars represent averages ± standard deviations (*n* = 4, *P* < 0.05*, *P* < 0.005**).

Oxylipins were measured in cerebella of frataxin knock-in/knockout (KIKO) deficient mice (29) using UPLC–MS/MS (Fig. 2). We quantified 33 arachidonic acid-derived oxylipins produced by COXs, lipoxygenases (LOXs) and cytochrome P450 (CYP). Twelve oxylipins were elevated in the KIKO mice (*P* > 0.05, *n* = 4) (Fig. 2), including the COX-derived prostanoids, thromboxanes, COX-side products 11 and 15 hydroxyeicosatetraenoic acids (HETE) (30), the CYP-derived epoxyeicosatrienoic acid (EET) and dihydroxyeicosatrienoic acid (DHET), and the 5LOX-derived HETE and ketoeicosatetraenoic acids (17,31,32). The results support the idea that frataxin deficiency in mice leads to increase in a global inflammatory response, including enhanced PG synthesis.

### Frataxin-deficient animal and cell models have increased COX2 expression

To understand the source of prostanoid elevation, we measured the protein concentration of both COX1 and COX2, the constitutive and inducible COXs, respectively (17,19,33,34). For this experiment, we utilized three different models of frataxin deficiency. The first model as described previously is the cerebella of KIKO mouse model compared with WT control. The second model is the cerebella of YG8 mouse model knocked out for endogenous frataxin gene and inserted with mutant human frataxin transgene comparing hemizygote with a single copy of transgene expressing 41.0% that of homozygote (15).

**Figure 3.**
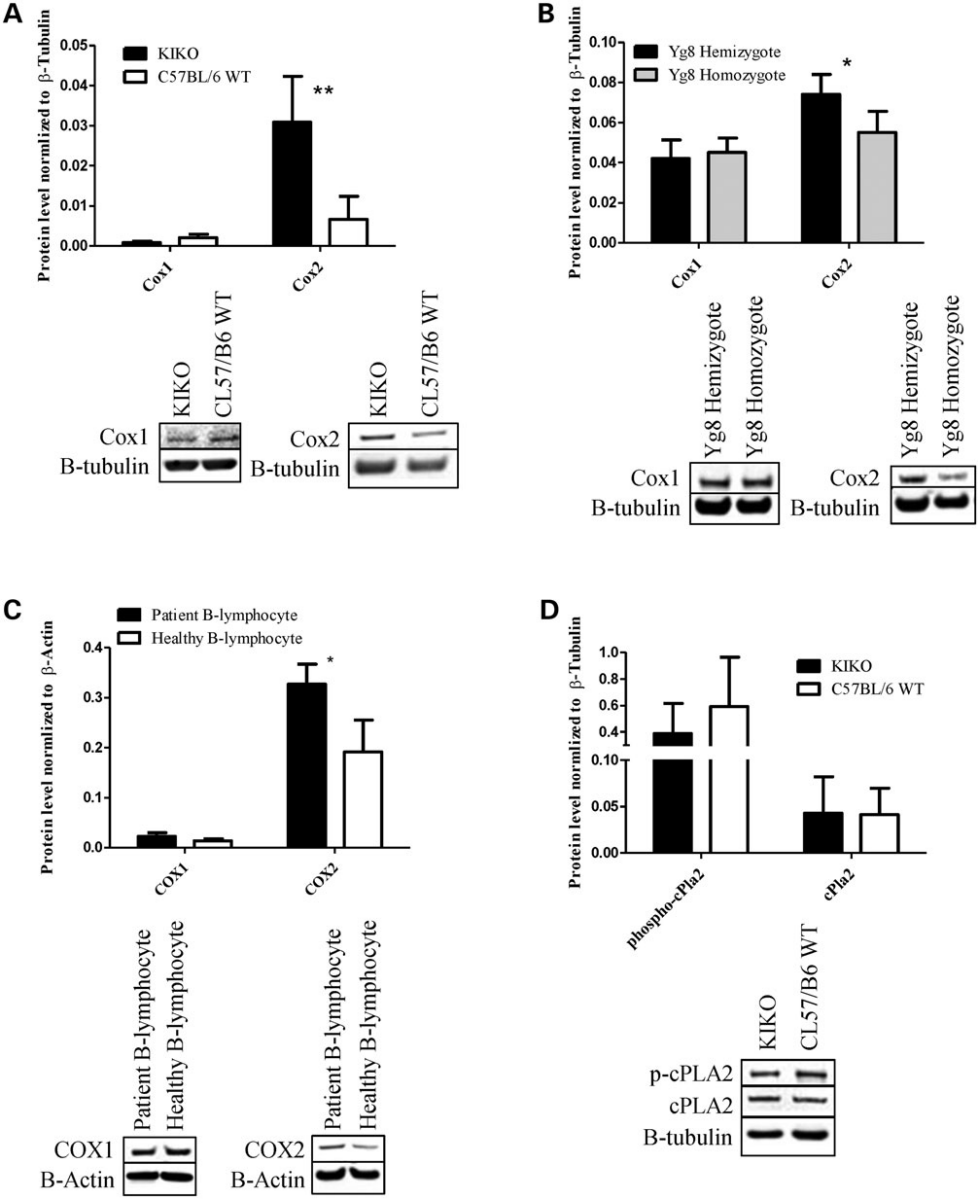
Frataxin-deficient models have increased expression of COX2 and no alterations in COX1 or cPLA2 expression. (**A** and **D**) Cerebellum of KIKO has 4.40-fold increased expression of Cox2 while no changes in Cox1 and cPla2 or the active phosphorylated cPLA2 were observed compared with C57Bl/6 WT. (**B**) Cerebellum of YG8 hemizygote showed 1.35-fold increased Cox2 expression and no change in Cox1 expression was observed compared with YG8 homozygote. (**C**) Patient B-lymphocytes had 1.71-fold increased expression of COX2 expression with no changes in COX1 expression compared with healthy B-lymphocytes. The KIKO and YG8 samples were normalized to β-tubulin and B-lymphocyte samples were normalized to β-actin. Bars represent averages ± standard deviations (*n* = 4, *P* < 0.05*, *P* < 0.005**).

COX 1 and 2 expression were also measured in human B-lymphocytes isolated from FRDA patients with 16.0% frataxin expression compared with healthy B-lymphocytes (*P* > 0.0020) (Fig. 1). Significantly increased protein levels of COX2 were observed in frataxin-deficient models, 4.16-fold in KIKO model (*P* > 0.0030, *n* = 4), 1.35-fold in YG8 model (*P* > 0.039, *n* = 4) and 1.92-fold in B-lymphocytes (*P* > 0.019, *n* = 4) while no changes in the expression levels of the constitutive isoform COX1 were detected (Fig. 3A–C).

Upstream of COX 1 and 2 is cytosolic phospholipase 2 (cPLA2), which hydrolyzes and releases arachidonic acid to be converted into PGG_2_ by COX. Thus, cPLA2 can potentially alter the prostanoid profile (19,33). We measured the protein level of both cPLA2 and its active form, phosphorylated at serine 505, and detected no significant difference between KIKO and WT mouse cerebellum (*n* = 4) (Fig. 3D). Taken together, these results support the notion that increased COX2 expression, rather than COX1 or cPLA2 activity, is the cause of increased prostanoid levels in frataxin-deficient models.

### Increased COX activity in frataxin-deficient mice and cells

**Figure 4.**
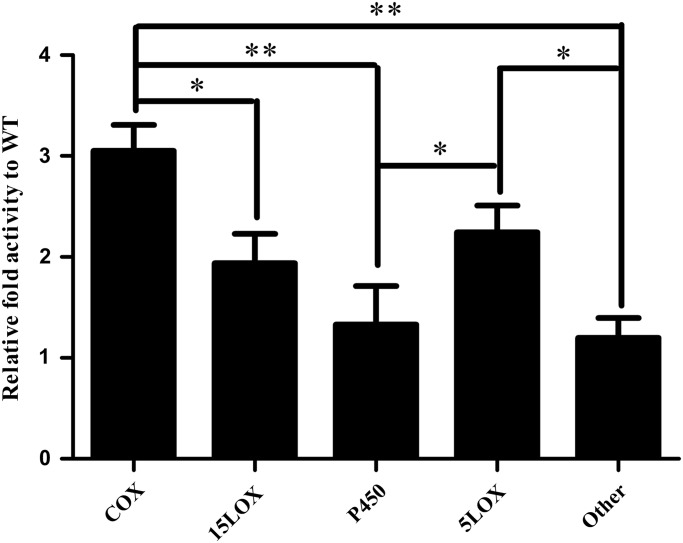
Frataxin-deficient KIKO mice have elevated COX metabolites normalized to free arachidonic acid relative to WT. All oxylipids downstream of arachidonic acid were normalized to total concentration of unbound arachidonic acid and fold change calculated relative to WT for the same metabolite. The COX metabolism (TXB2, PGF1a, PGE_2_, PGD_2_, PGJ_2_, PGF_2_a and 11 HETE) was 3.05-fold higher than WT, 15LOX metabolism (15 HETE) was 1.94-fold higher than WT, 5LOX metabolism (5 HETE) was 2.24-fold higher than WT, CYP metabolism (14,15DHET and 11,12-DHET) was 1.33-fold higher than WT, other arachidonate metabolism (8,15DHET, 5,15DHET, 9HETE and 8HETE) was 1.20-fold higher than WT. Bars represent averages ± standard error mean (*n* = 4, *P* < 0.05*, *P* < 0.00005**).

In order for an increase of COX expression to impact prostanoid concentrations, the increase must impact COX activity. Thus we analyzed the arachidonate metabolites of the KIKO cerebellar tissue normalized to both total concentration of unbound arachidonic acid and also to WT to identify the relative activity of downstream enzymes. In mutants the COX metabolites were 3.05-fold higher than WT, 15LOX metabolites were 1.94-fold higher than WT, 5LOX metabolites were 2.24-fold higher than WT, CYP metabolites were 1.33-fold higher than WT, and other arachidonic acid metabolites were 1.20-fold higher than WT (*n* = 4) (Fig. 4). COX metabolites were significantly higher than those of 15LOX (*P* > 0.02), CYP (*P* > 1.54 × 10^−5^) and other arachidonate metabolites (*P* > 1.13 × 10^−6^). We also observed significant elevation of 5LOX metabolites compared with CYP (*P* > 0.03) and other arachidonate metabolites (*P* > 0.018) (*n* = 4) (Fig. 4).

**Figure 5.**
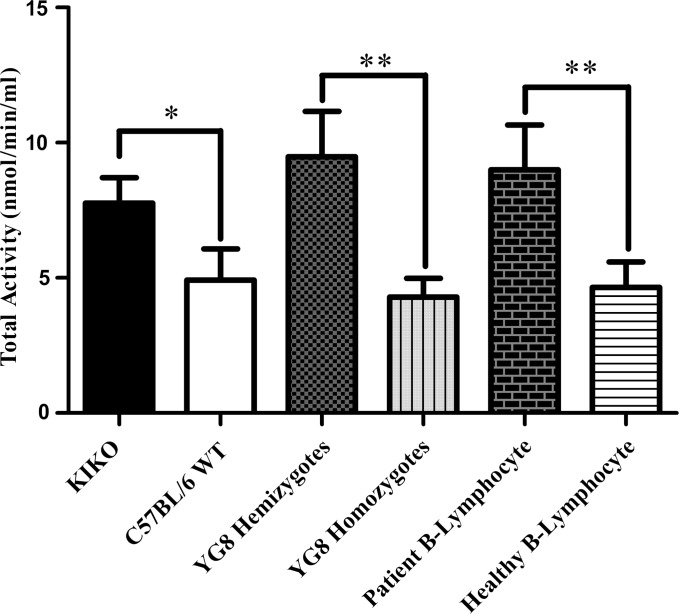
Frataxin-deficient models have increased total COX activity of cerebellar or B-lypmphocyte lysate. All FRDA models, KIKO, YG8 and patient B-lymphocytes showed 1.58-, 2.21- and 1.94-fold increase in COX activity respective to each model's control. Bars represent averages ± standard deviations (*n* = 3–4, *P* < 0.05*, *P* < 0.01**).

We also performed an ELISA-based activity assay measuring the conversion rate of hydrolyzed arachidonic acid to PGG_2_, a precursor to PGH_2_. Comparing the cerebellum of frataxin-deficient mouse KIKO and YG8, and patient B-lymphocyte to either WT or healthy controls, we found significant elevation of COX activity when normalized to indomethacin-inhibited samples. KIKO hemizygote showed 1.58-fold activity (*P* > 0.030, *n* = 3), YG8 hemizygote showed 2.21-fold activity (*P* > 0.0012, *n* = 4) and patient B-lymphocyte showed 1.92-fold activity (*P* > 0.0069, *n* = 4) relative to their respective controls (Fig. 5). Taken together with the immunoblot results, the likely driving force of increased prostanoid concentration in the frataxin-deficient models is the overexpression and the subsequent increased activity of COX2.

### Elevated CREB and AP1 transcription factors explain COX2 overexpression in frataxin-deficient cells

**Figure 6.**
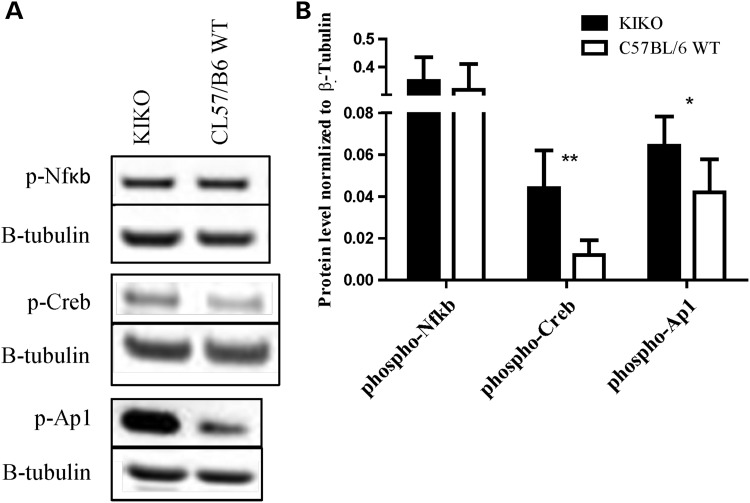
Cerebellum of frataxin-deficient KIKO mouse model has increased activation of transcription factors CREB and AP1. (**A** and **B**) KIKO shows 3.67- and 1.52-fold increase of CREB and AP1 activation, respectively. Activation is marked by phosphorylation. Bars represent averages ± standard deviations (*n* = 6, *P* < 0.05*, *P* < 0.005**).

Expression of the COX2 gene is driven by multiple positive factors, including NFκB, CREB and AP1. To understand whether or how frataxin deficiency was driving an intrinsic increase in COX2 gene activity, we used antibody specific to activated transcription factors, phosphorylated NFκB (ser536), phosphorylated CREB (ser133) and phosphorylated AP1 (ser63) known to positively regulate COX2 expression, in the cerebella of KIKO mouse (21–23). We found no difference in the activation of NFκB (*n* = 6) but identified a 3.67-fold increase in CREB activation (*P* > 0.0024, *n* = 6) and 1.52-fold increase in AP1 activation (*P* > 0.028, *n* = 6) (Fig. 6A and B). This suggests that in the context of the frataxin-deficient cerebellum, CREB and AP1 increase drive the COX2 overexpression and increased PG production.

### Increased microglial activation in lipopolysaccharide induced frataxin-deficient mouse cerebellum

**Figure 7.**
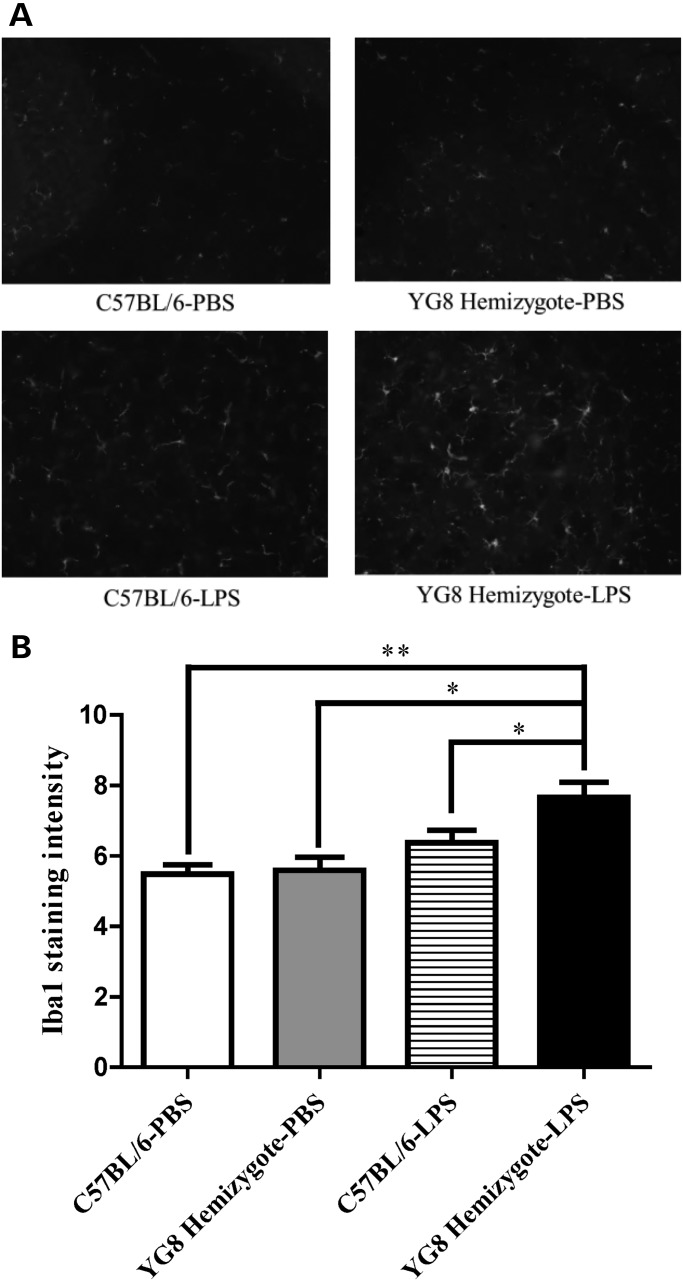
Frataxin-deficient YG8 hemizygote mouse cerebellum shows increased inducibility of inflammation with LPS treatment compared with WT. (**A**) Immunohistochemistry of frataxin-deficient YG8 hemizygote and WT cerebellar section with antibody specific to Iba1. The LPS treatment has increased the protein concentration of Iba1 in mutants 1.37-fold compared with WT which increased 1.16-fold from similar baseline expression in the PBS treatment. (**B**) Quantification of immunohistochemistry. Bars represent averages ± standard deviations (*n* = 4, *P* < 0.05*, *P* < 0.005**).

To understand whether COX2 overexpression and increased prostanoids were having an impact on neuroinflammation in the frataxin-deficient context, we measured ionized calcium-binding adapter molecule 1 (Iba1), a known marker of microglial neuroinflammation in the cerebella of YG8 mice, in the context of the experimental inflammogen lipopolysaccharide (LPS) (20,35). The treatment of WT with LPS did not significantly increase the expression of Iba1 in cerebellum with 1.16-fold elevation, but the same treatment in the YG8 hemizygotes had significantly increased the Iba1 expression by 1.37-fold (*P* > 0.0096, *n* = 4). Accounting for baseline Iba1 expression in WT and YG8 hemizygote cerebellum treated with PBS, YG8 hemizygotes had 17% increase in induction of Iba1 by LPS compared with WT (Fig. 7A and B)]. Thus, frataxin deficiency increased the reactivity of iba 1 staining microglia in the context of LPS treatment relative to WT.

## DISCUSSION

FRDA is an autosomal recessive disease characterized by the degeneration of the dorsal root ganglion and cerebellum (2,4). Clinical symptoms of FRDA include gait ataxia affecting the motor coordination, weakness and atrophy of the extremities, and loss of lower-limb tendon reflexes (5,36,37). The disease state is caused by the reduced expression of a nuclear encoded gene, frataxin mediated by the repeat expansion of GAA within the first intron of the gene altering the epigenetic control of the gene transcription (6,7,38). However, the pathomechanism by which frataxin deficiency causes neuro- and cardio-degeneration is not clear. We previously demonstrated inflammatory consequences of frataxin knockdown in Schwann tissue culture cells (20), and also induction of inflammatory transcripts and suppression of antioxidant and NRF2 anti-inflammatory molecules in a mouse model of FRDA (14). The reduced capacity of NRF2 and thiol antioxidant proteins (Glrx1, Gstm1 and Prdx3) is thought to be involved in the production of ROS in the cerebellar tissue and possibly in the lymphocyte cells by suppressing the cell's capacity to maintain homeostatic anti-oxidative capability. The suppression of the anti-inflammatory factor NRF2, and the elevated expression of the pro-inflammatory prostaglandin D_2_ synthase (PTGDS) transcript motivated a search for changes in inflammatory mediators in brains of a Friedreich's animal model.

Four PGs and TXB2 were elevated significantly in KIKO mice over controls. Of the elevated eicosanoids, 8 of 12 were the products of COX-mediated conversion of arachidonic acid, supporting the notion that the COX activity could play a role (Fig. 2A and B) (17,31,32). We also observed elevation of LOX and CYP metabolized products, which have been previously reported and may be the result of parallel pathway activated by oxidative stress (24,26,28,39). Many of the non-COX metabolite mass spectrometry hits including 11,12EET, 15HETrE and 5HETE have been reported to have anti-inflammatory effects in response to excess production of ROS (40–42). Interestingly, 15LOX and COX mediated metabolite 15HETE is believed to be involved in the progression of Alzheimer's disease and the reduction of 15HETE in Alzheimer's model shows evidence of decreased oxidative stress (32,43). Furthermore, increase of specific CYPs may also contribute to the compounding problem of oxidative stress by synthesizing ROS but this effect has yet to be investigated in the context of FRDA (44). Similar rises in prostanoid synthesis have been reported to have neuroinflammatory effects leading to neurodegeneration (45). PGs including PGE_2_ and 15d-PGJ_2_ have been associated with pathogenesis of neurodegenerative diseases such as Alzheimer's disease (24,25), Parkinson's disease (26) and Amyotrophic lateral sclerosis (27,28).

We also looked into more general lipid composition analysis to determine whether frataxin deficiency simply elevates lipid synthesis nonspecifically. Mass spectrometry analysis revealed only minor elevation of total fatty acid in the KIKO mice compared with those of WT, but the major elevation was observed in the COX-mediated oxylipid metabolism, supported by slight decrease in the concentration of linoleic acid (C18:2n6) in the KIKO mice (Supplementary Material, Fig. S1). The frataxin-deficient KIKO mouse has increased concentration of prostanoids synthesized primarily by COX, and this elevation may contribute to increased inflammation, which may ultimately lead to degeneration of the dorsal root ganglion and cerebellum.

The action of both COX and cPLA2 play key roles in the synthesis of prostanoids. Initially, cPLA2 regulates the availability of free arachidonic acid by hydrolysis and cleavage from the phospholipid bilayer followed by COX-mediated conversion of arachidonic acid to the prostanoids (17–19,27,46). In this study we showed by immunoblot analysis of KIKO and YG8 cerebellum as well as B-lymphocyte cells, the likely cause of prostanoid concentration elevation observed by mass spectrometry (Fig. 2A and B) resulted from the overexpression of the inducible COX isoform, COX2 rather than the constitutive isoform, COX1 (19,33,34) (Fig. 3A–C). We identified no alterations in protein concentration of cPLA and phospho- cPLA in the KIKO mice, indicating that cPLA2 does not cause the increased prostanoid burden in mutant mice (Fig. 3D) (46).

Total COX activity assays also supported the notion that COX2 overexpression has increased the conversion rate of arachidonic acid to downstream prostanoids (Fig. 5). These data are supported by arachidonate metabolite analysis, indicating that the more of the unbound arachidonic acid is being converted into downstream metabolites of COX than any other enzyme (Fig. 4). Both 15- and 5-LOX activity appear to be elevated compared with WT, which may be a consequence of basally elevated ROS in the frataxin-deficient state (32,42). Taken together, the reduction in frataxin expression has increased the expression of COX2 increasing the synthesis of prostanoids. Given the increased prostanoid profile in the KIKO mouse model, one could consider the reversal of COX2 overexpression by NSAIDs, but this was beyond the scope of this work, but is a future prospect.

**Figure 8.**
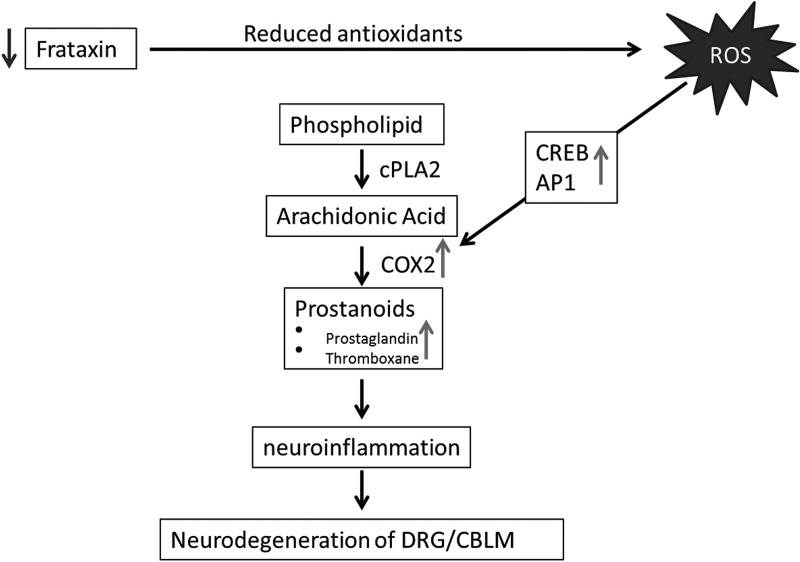
Model for frataxin deficiency with elevation of prostanoids leading to neurodegeneration of dorsal root ganglion (DRG) and cerebellum (CBLM).

COX2 expression is regulated by three transcription factors, NFκB, CREB and AP1, and of these, we have found increased activation of CREB and AP1 (22). The CREB and AP1 activation was 3.67 and 1.52-fold higher in the KIKO mice, respectively, indicating multiple pathway activation of COX2 transcription and the greater role of CREB in COX2 activation (Fig. 6A and B). Both CREB and AP1 transcription factors have been reported to be activated by cytokine pathways mediated through the presence of ROS (23,47). Interestingly, the chronic elevation of oxidative stress in FRDA patients and animal models may be a contributing factor in the activation of cytokines responsible for COX2 overexpression (8–12), suggesting the mechanism fxn-deficiency->ROS->cytokines->CREB/AP1->COX2->iba1/microglial activation->neuroinflammation->neurodegeneration (Fig. 8). It cannot be ruled out that the dysregulation of NRF2 in the frataxin-deficient mice (14) (Supplementary Material, Fig. S3) is uninvolved in the overexpression of COX2 as it has previously been shown to be the case in the brain of Nrf2^−/−^ mice and plays a role in further elevating inflammatory state of frataxin-deficient mice (48).

One question that arises from these data in Friedreich's patient cells and two mouse models is whether evidence of inflammation is also observed in either living Friedreich's patients or autoptic tissue. There is clear evidence of inflammation in Friedreich's autoptic hearts marked by cluster of differentiation 68 (CD68) positive cells (49); CD68 is a marker of microglial activation. Microglia are more activated in brains of LPS-stimulated mutant mice than in controls, consistent with a frataxin-dependent increase in microglial activation in these cerebellar samples (Fig. 7). These data suggest that frataxin deficiency leads to microglial activation in Friedreich's hearts and mouse model brains. We see increased production of oxylipins in cerebella of mutant mice, and the median increase was mild, about 56% (Supplementary Material, Fig. S2). These mice also have a mild neurobehavioral phenotype, and a mild amount of neurodegeneration. Similarly, we do not see a difference in iba1-positive microglia at baseline; the effect is once again mild and is only visible and significant when provoked with an experimental inflammogen (LPS). Thus, either as a cause or effect of the mild rise in oxylipins, there is more ‘inducibility of inflammation’ in the mutant mice. Furthermore, active microglia has been shown to produce ROS, mainly nitrogen oxide further exacerbating the problem (44). Telomere length, an indirect marker of inflammation, was also measured in Friedreich's patient plasma correlating disease duration with reduction in telomere lengths (50–52). These cases represent the need for further research concerning inflammation as a possible pathomechanism of FRDA.

In conclusion, we have shown in cerebellum of frataxin-deficient mouse models KIKO and YG8, and patient derived lymphocytes, that frataxin deficiency leads to increased activation of transcription factors CREB and AP1 mediating the overexpression of COX2 subsequently elevating concentrations of multiple prostanoids, and that cerebella of such mice are more sensitive to a neuroinflammatory stimulus (Fig. 7). Further studies are necessary to understand the extent to which neurodegeneration of dorsal root ganglion and cerebellum in FRDA is caused by prostanoid alteration, and if the causal regulatory changes are due to chronic elevation of ROS. If prostanoid synthesis is involved in the pathogenesis of FRDA, anti-inflammatory treatments targeting COX, or upstream transcription factors CREB and AP1 may open new avenues for FRDA treatment.

## MATERIAL AND METHODS

### Cell culture

Human B-lymphocyte cells; GM16220, GM16197, GM15851, GM15850, GM04079, GM00607, GM00333 and GM00130 (Coriell Institute) were maintained at 37°C in humidified atmosphere with 5% CO_2_. DMEM (Cellgro) supplemented with 15% fetal bovine serum (JR-Scientific), 2 mm sodium pyruvate (Sigma-Aldrich), 1× antibiotic-antimycotic, 1× MEM non-essential amino acids (Gibco), and 50 mg/ml uridine (MP-Biomedicals) was used to grow cells.

### Mouse models and dissection

Human transgenic frataxin-deficient mice YG8 (15) (YG8Pook/J; Jackson Laboratory, Sacramento, CA, USA) and endogenous frataxin-deficient KIKO mice [a kind gift from Dr. Pandolfo (29)] were housed in a vivarium maintained at 22–24°C and 40–60% relative humidity with a 12-h light/12-h dark cycle. All experimental procedures were approved by the University of California Institutional Animal Care and Use Committee.

The mice were decapitated and cerebellums were immediately removed and then flash frozen with liquid nitrogen. Samples were stored in −80°C until utilized for mass spectrometry, quantitative PCR, western blot and COX activity assay.

### RNA extraction and quantitative RT–PCR

Total RNA was extracted from cerebellar tissue of frataxin-deficient KIKO and YG8 mice as well as B-lymphocyte cells using RNeasy plus mini kit (Qiagen) following manufacturer's instruction and RNA quantity measured by NanoDrop 2000c Spectrophotometer (Thermo Scientific).

Quantitative PCR was performed using the Superscript III One Step kit (Invitrogen) per manufacturer's instruction in a Roche Lightcycler 480 (Roche Diagnostics). Primer sequences are β-actin Forward GCCAACACAGTGCTGTCTGG, β-actin Reverse CTGCTTGCTGATCCACATCTGC, YG8 FXN Forward CTGGCTATCTTCTCCATCCAG, YG8 FXN Reverse AGCATCTTTTCCGGAATAGGC, KIKO Fxn Forward GATCAACAAGCAGACCCCAAA, KIKO Fxn Reverse AGGCCAATGAAGACAAGTCCA, B-Lymphocyte FXN Forward ATCTTCTCCATCCAGTGGACCT and B-Lymphocyte FXN Reverse GCTGGGCATCAAGCATCTTTT. The data were analyzed by delta delta CT method.

### Protein extraction and western blot analysis

Mouse cerebellar tissues and B-lymphocyte cell pellets were homogenized with a cell lysis buffer (Cell Signaling) with Halt phosphatase inhibitor (Thermo-Fisher), complete protease inhibitors (Roche) and PSMF (Sigma-Aldrich). Thirty micrograms of lysates were loaded into 4–12% Bis–Tris gels (Invitrogen). Electrophoresis was carried out according to the manufacturer's recommendations. Following electrophoresis, the proteins were transferred to nitrocellulose membranes by the iBlot device (Invitrogen), blocked with an Odyssey blocking buffer (LI-COR Biotechnology) for 1 h. Membranes were incubated overnight with the following primary antibodies in blocking buffer: COX1 (ab133319 for human, ab133319 for mouse; Abcam), COX2 (ab62331 for human, ab6665 for mouse; Abcam), phospho-CREB (4095), phospho-cJun/AP1 (5464), phospho-NFκB (4025; Cell signaling), cPLA2 (sc-454), phospho-cPLA2 (sc-34391; Santa Cruz Biotech, Santa Cruz, CA, USA), β-actin (A5441; Sigma-Aldrich), and β-tubulin (DSHB-E7; DSHB, Iowa). Subsequently, the membranes were incubated with a corresponding pair of IRDye 680CW and IRDye 800CW-coupled secondary antibodies (LI-COR). Proteins were visualized with the Odyssey infrared imager and software (LI-COR) according the manufacturer's instruction.

### LPS administration and immunostaining of Iba1

Female YG8 mice or C57BL/6 mice were intraperitoneally injected with a single dose of LPS (5 mg/kg). Mice were perfused with 4% paraformaldehyde in 0.1 m PBS at 24 h after LPS injection. The animals were anesthetized and then perfused with 4% paraformaldehyde in 0.1 m PBS (pH 7.4) and cryoprotected in 30% sucrose in 0.1 m PBS. 15 µm coronal brain sections from cerebellum were cut by cryostat. Representative sections were stained with Iba1 antibody (1:100; Wako, VA, USA) followed by a fluorescent secondary antibody (Invitrogen, NY, USA). The images of Iba1 fluorescent immunostaining were taken with Nikon camera (Tokyo, Japan). Fluorescent intensity from six fields of each animal was measured by Image J software.

### COX activity

Total COX activity was measured with COX Fluorescent Activity Assay Kit (Cayman Chemical, Ann Arbor, MI, USA) according to manufacturer's instruction normalized to samples incubated with Indomethacin (Sigma-Aldrich).

### Tissue extraction

Frozen mouse cerebellar samples (20–100 mg) were pulverized on dry ice, after mixing with 5 μl BHT/EDTA (0.2 mg/ml in 1:1 methanol/water), and 30 µl methanolic surrogate solutions containing a suite of labeled surrogates to quantify cholesteryl ester, triglyceride, phospholipid, free fatty acid, prostanoid, thromboid, diol, alcohol, epoxide and sphingoid bases. Samples were introduced to two pre-cleaned stainless steel balls and vortexed with 500 µl methanol for 3 min. Samples were pelleted by centrifugation for 5 min at 10 000 rcf, 10°C, and the supernatant was isolated. Residues were mixed with 350 µl 2-propanol, homogenized and pelleted and the supernatant was combined with the primary methanolic extract. The residue was extracted a third time with 350 µl cyclohexane, followed by mixing and supernatant isolation and combination with the other organic isolates. Solvents were removed by vacuum evaporation for 60 min without heating and reconstituted in 100 µl toluene and mixed with 100 µl methanol. A 10 µl aliquot of the extract was isolated for fatty acid analyses while 180 µl were evaporated to dryness and reconstituted in 100 µl methanol containing 1-cyclohexyl-3-ureido-dodecanoic acid (CUDA), filtered at with Amicon^®^ Ultrafree-MC Durapore PVDF 0.1 µm filters (Millipore, Billerica, MA, USA) and used for the quantification of oxylipins.

### UPLC-tandem mass spectrometry

Analytes were separated by reverse phase ultra-performance liquid chromatograph with a 1.7 µm Acquity BEH column (Waters, Milford, MA, USA) using a 16 min gradient (Solvent A = 0.1% acetic acid; Solvent B = 90:10 v/v acetonitrile/isopropanol), detected on an API 4000 QTrap (AB Sciex, Framingham, MA, USA) by multiple reaction monitoring after negative mode electrospray ionization, and quantified against 6 point calibration curves using internal standard methodologies as previously reported (doi:10.1371/journal.pone.0089393). Data reporting criteria include: data >3:1 signal to noise; the relative contribution of background from method blanks is <25% of signal; values are within the calibrated linear range.

### Data analysis

Quantified results were manually curated to confirm accurate integrations. NEFA results for a single sample (ID#1) were statistical outliers by the Dixon's Q-test and were excluded. All missing values were computationally imputed using a probabilistic principal components analysis (PCA). Data were transformed to normality prior to statistical analyses using the procedures of Box and Cox. Differences in means were determined using 2-tailed *T*-tests or Mann–Whitney *U*-tests if normality was not achieved. The effects of frataxin gene on lipid profiles were assessed using multivariate analyses including correlations, cluster analyses and principal components analyses. Statistical operations were performed within Microsoft Excel. Multivariate and non-parametric statistics were performed using the Excel Add-In imDEV v1.4.2 (http://sourceforge.net/apps/mediawiki/imdev/index.php?title=Main_Page), which provides a graphical user interface to R-Programing language statistical packages (doi: 10.1093/bioinformatics/bts439).

## SUPPLEMENTARY MATERIAL

Supplementary Material is available at *HMG* online.

## FUNDING

The study was supported by NIH grants NS077777, EY012245 and AG025532 to G.A.C., and USDA-ARS Intramural Projects 5306-51530-019-00D and 1 U24 DK097154-01 to J.W.N. Funding to pay the Open Access publication charges for this article was provided by the NIH.

## Supplementary Material

Supplementary Data
