# Supplementary material for: Frataxin deficiency increases cyclooxygenase 2 and prostaglandins in cell and animal models of Friedreich's ataxia
Source: Hum Mol Genet. 2014 Aug 7;23(25):6838–47. doi: 10.1093/hmg/ddu407 (PMC4245045; doi:10.1093/hmg/ddu407)
Supplement: Supplementary Data [file supp_23_25_6838__index.html]

Frataxin deficiency increases cyclooxygenase 2 and prostaglandins in cell and animal models of Friedreich's ataxia — Frataxin deficiency increases cyclooxygenase 2 and prostaglandins in cell and animal models of Friedreich's ataxia — Supplementary Data 

# Frataxin deficiency increases cyclooxygenase 2 and prostaglandins in cell and animal models of Friedreich's ataxia

## Supplementary Data

Supplementary Data

**Files in this Data Supplement:**

- Supplementary Data - Docx file
