# Supplementary material for: Frataxin deficiency increases cyclooxygenase 2 and prostaglandins in cell and animal models of Friedreich's ataxia
Source: Hum Mol Genet. 2014 Aug 7;23(25):6838–47. doi: 10.1093/hmg/ddu407 (PMC4245045; doi:10.1093/hmg/ddu407)
Supplement: Supplementary Data [file supp_ddu407_ddu407supp.docx]

**Supplementary Figures**


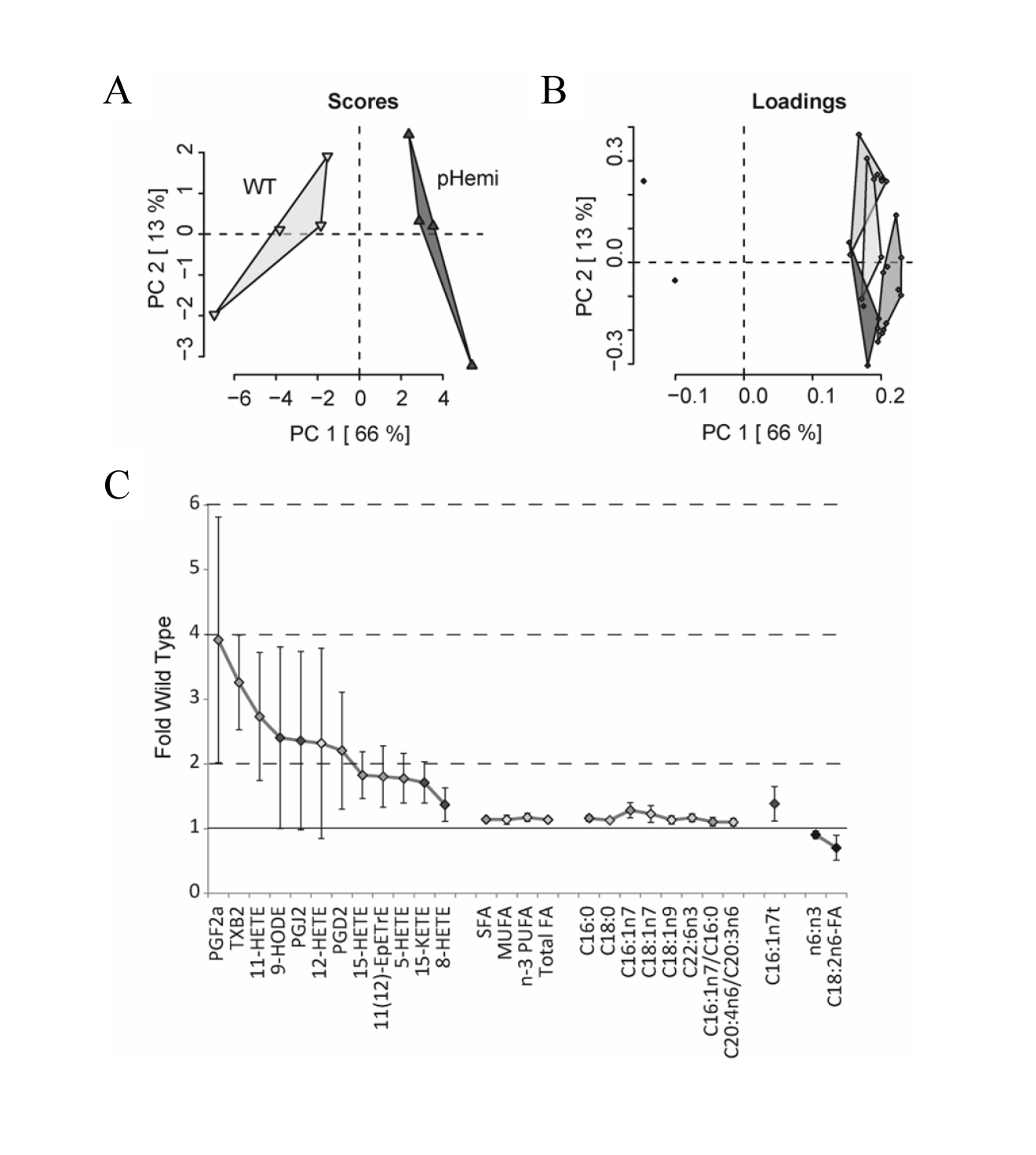
Supplementary Fig S1. Lipid composition alterations as a result of frataxin deficiency are driven by COX mediated metabolism. (A-B) Principle component analysis plot shows the genotypic difference of KIKO and WT mice are driving the changes in fatty acid composition. (C) Relative lipid concentrations of fatty acids with varying saturation are listed along with those metabolized by COX. Slight increase is indicated for the non-COX mediated fatty acids shown between SFA and C20:4n6/C20:3n6, while significant elevation of lipids are observed for COX mediated fatty acid shown left of 8HETE. C18:2n6 and n6:n3 are upstream of COX metabolized lipids and are shown to be relatively lower in frataxin deficient KIKO mice. SFA-saturated fatty acid, MUFA- Monounsaturated fat, n-3PUFA-polysaturated fatty acid, FA-fatty acid. Bars represent averages±standard mean error (n=4).





Supplementary Fig S2. Median fold change in eicosanoid concentration is mild in the frataxin deficent cerebellar tissue. Median fold change is 1.56 while the maximum and minimum are 3.92 and 0.69 respectively in 33 eicosanoid parameters (n=4).


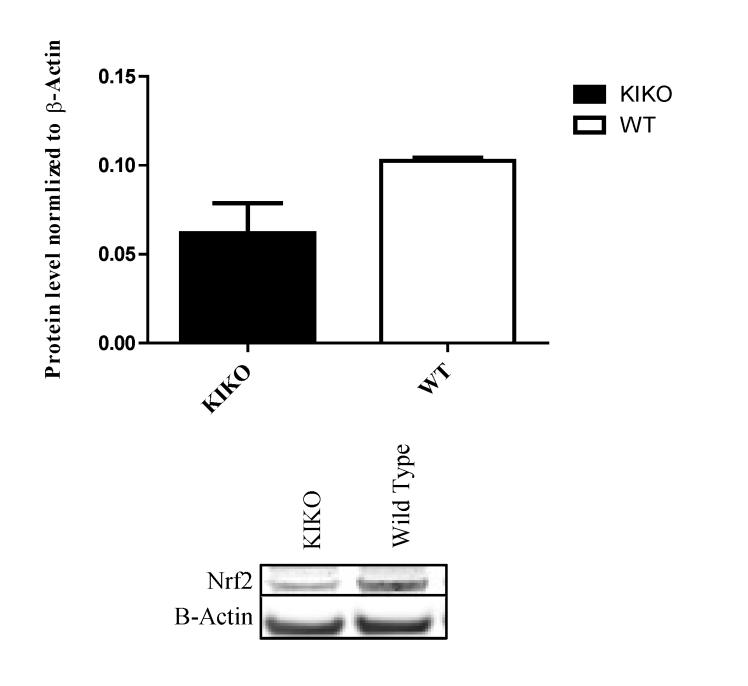


Supplementary Fig S3. Nrf2 protein expression is diminished in KIKO mouse compared to that of wild type. The average expression of Nrf2 in KIKO mice is 0.062 while wild type was 0.103 when normalized to β-actin. Bars represent averages±standard mean error (n=2).

| Lipid name | p-value |
| --- | --- |
| TXB2 | 0.007 |
| PGD2 | 0.071 |
| PGF2a | 0.054 |
| PGJ2 | 0.105 |
| 11-HETE | 0.036 |
| 5-HETE | 0.032 |
| 15-KETE | 0.015 |
| C16:0 | 0.031 |
| C18:0 | 0.026 |
| SFA | 0.071 |
| C16:1n7 | 0.011 |
| C22:6n3 | 0.015 |
| n6:n3 | 0.047 |
| C16:1n7t | 0.058 |
| n-3PUFA | 0.012 |
| TotalFA | 0.092 |
| C18:1n7 | 0.113 |
| C18:1n9 | 0.152 |
| MUFA | 0.174 |
| 11(12)-EpETrE | 0.034 |
| C18:2n6-FA | 0.068 |
| 9-HODE | 0.14 |
| 15-HETE | 0.015 |
| 12-HETE | 0.153 |
| 8-HETE | 0.169 |
| C20:4n6.C20:3n6 | 0.028 |
| C16:1n7.C16:0 | 0.045 |

Supplementary Table T1. P-values of supplementary Fig S1.
